# Supplementary material for: High-Frequency Transcranial Random Noise Stimulation over the Left Prefrontal Cortex Increases Resting-State EEG Frontal Alpha Asymmetry in Patients with Schizophrenia
Source: J Pers Med. 2022 Oct 7;12(10):1667. doi: 10.3390/jpm12101667 (PMC9604798; doi:10.3390/jpm12101667)
Supplement: Supplementary file 1 [file jpm-12-01667-s001.zip › jpm-1910079-supplementary.pdf]

Supplementary materials

High-frequency transcranial random noise stimulation over the left prefrontal cortex increases resting-state EEG frontal alpha asymmetry in patients with schizophrenia

#Corresponding author: Hsin-An Chang, M.D. (Professor and Attending Psychiatrist) Department of Psychiatry, Tri-Service General Hospital, No. 325, Cheng-Kung Road, Sec. 2, Nei-Hu District, Taipei, 114, Taiwan

Tel/Fax: 011-886-2-8792-7220 / 011-886-2-8792-7221

E-mail: chang.ha@mail.ndmctsgh.edu.tw

This supplementary information contains:

Supplementary figure and tables

1. Supplementary figure

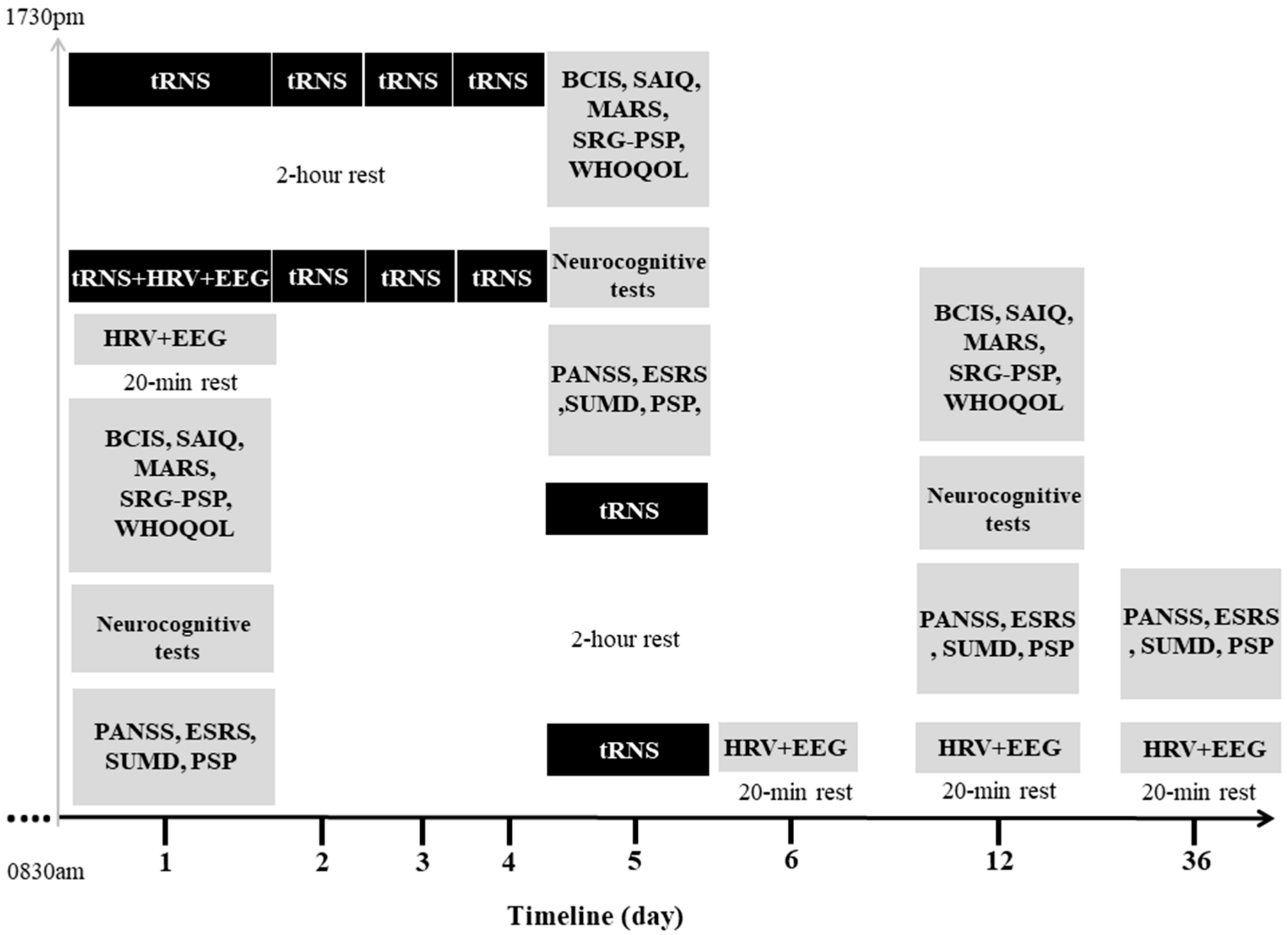

Figure S1. The flow chart of all the measurements conducted at baseline, after 10-session stimulation, and at the follow-up visits. PANSS, Positive and Negative Syndrome Scale; ESRS, Extrapyramidal Symptoms Rating Scale; SUMD, the abbreviated version of the Scale to Assess Unawareness in Mental Disorder in schizophrenia; PSP, the Taiwanese version of the Personal and Social Performance scale; BCIS, the Taiwanese version of the Beck's Cognitive Insight Scale; SAIQ, the Taiwanese version of the Self-Appraisal of Illness Questionnaire; MARS, the Taiwanese version of Medication Adherence Rating Scale; SRG-PSP, Self-reported version of the graphic Personal and Social Performance scale; WHOQOL, World Health Organization Quality of Life-BREF; HRV, heart rate variability; EEG, electroencephalography; tRNS, high-frequency transcranial random noise stimulation over the lateral prefrontal cortex.

2. Supplementary tables

Table S1. The changes in the severity of negative symptoms as a whole, Avolition-Apathy and Expressive Deficit domains of negative symptoms, disorganization symptoms and antipsychotic-induced extrapyramidal motor symptoms between hf-tRNS group and sham condition.

	At the end of stimulation			One-week follow-up			One-month follow-up		
	Active	Sham	p value	Active	Sham	p value	Active	Sham	p value
FSNS score	-3.76 ± 2.17	-0.33 ± 0.49	<.001	-3.65 ± 2.64	0.06 ± 1.30	<.001	-3.76 ± 2.81	0.06 ± 1.30	<.001
AA domain score	-1.71 ± 0.99	-0.17 ± 0.38	<.001	-1.65 ± 1.27	-0.11 ± 0.58	<.001	-1.65 ± 1.37	-0.17 ± 0.71	<.001
EXP domain score	-3.00 ± 1.66	-0.22 ± 0.73	<.001	-3.06 ± 2.08	0.28 ± 1.45	<.001	-3.29± 2.28	0.17 ± 1.47	<.001
Disorganization dimension score	-1.53 ± 1.07	0.00 ± 0.34	<.001	-1.47 ± 1.01	-0.06 ± 0.42	<.001	-1.47 ± 1.01	-0.07 ± 0.62	<.001
ESRS score	-3.12 ± 2.78	-0.33 ± 0.69	<.001	-2.76 ± 2.56	-0.22 ± 0.43	<.001	-1.53 ± 1.59	-0.22 ± 0.43	.004

Abbreviations: FSNS, Positive and Negative Syndrome Scale Factor Score for Negative Symptoms; AA, Avolition-Apathy; EXP, Expressive Deficit; ESRS, Extrapyramidal Symptoms Rating Scale.

Table S2. Associations between the daily doses of concomitant medications and baseline electroencephalographic frontal alpha asymmetry (FAA).

	F4-F3 FAA		Fp2-Fp1 FAA		F8-F7 FAA	
Daily doses of concomitant medications	r	p	r	p	r	p
Daily dose of antipsychotic medications[a]	0.20	0.26	-0.10	0.59	0.06	0.73
Daily dose of anticholinergic antiparkinsonian medications[b]	0.14	0.42	0.17	0.32	0.18	0.31
Daily dose of sedative-hypnotics[c]	0.39	0.02	0.29	0.09	0.12	0.50

[a]The daily dose of antipsychotic medications was converted to chlorpromazine equivalent

[b]The daily dose of anticholinergic antiparkinsonian medications was converted to biperiden equivalent

[c]The daily dose of sedative-hypnotics was converted to diazepam equivalent

P values that reach the corrected significance level (false discovery rate method) are in bold.
